# Energy-Efficient Data Collection Method for Sensor Networks by Integrating Asymmetric Communication and Wake-Up Radio

**DOI:** 10.3390/s18041121

**Published:** 2018-04-06

**Authors:** Masanari Iwata, Suhua Tang, Sadao Obana

**Affiliations:** Graduate School of Informatics and Engineering, The University of Electro-Communications, Chofu, Tokyo 182-8585, Japan; m.iwata@uec.ac.jp (M.I.); shtang@uec.ac.jp (S.T.)

**Keywords:** sensor network, energy efficient, mobile sink, asymmetric communication, wake-up radio

## Abstract

In large-scale wireless sensor networks (WSNs), nodes close to sink nodes consume energy more quickly than other nodes due to packet forwarding. A mobile sink is a good solution to this issue, although it causes two new problems to nodes: (i) overhead of updating routing information; and (ii) increased operating time due to aperiodic query. To solve these problems, this paper proposes an energy-efficient data collection method, Sink-based Centralized transmission Scheduling (SC-Sched), by integrating asymmetric communication and wake-up radio. Specifically, each node is equipped with a low-power wake-up receiver. The sink node determines transmission scheduling, and transmits a wake-up message using a large transmission power, directly activating a pair of nodes simultaneously which will communicate with a normal transmission power. This paper further investigates how to deal with frame loss caused by fading and how to mitigate the impact of the wake-up latency of communication modules. Simulation evaluations confirm that using multiple channels effectively reduces data collection time and SC-Sched works well with a mobile sink. Compared with the conventional duty-cycling method, SC-Sched greatly reduces total energy consumption and improves the network lifetime by 7.47 times in a WSN with 4 data collection points and 300 sensor nodes.

## 1. Introduction

Wireless Sensor Networks (WSNs) have been extensively investigated for various fields, e.g., agriculture and environmental sensing [1,2]. In wild environments, sensor nodes, despite their limited battery capacity, are not attended to (no recharge, no battery exchange) any more once deployed. Therefore, the question of how to design energy-efficient protocols to extend network lifetime is a big challenge. Because of severe attenuation in wireless signal propagation, multi-hop communication is generally used to collect sensor data from sensor nodes to a sink node in large-scale WSNs. However, in this way, sensor nodes close to the sink node consume energy more quickly than other nodes due to packet forwarding, which may lead to network disconnection. Therefore, the energy efficiency of relay nodes is an important factor for extending the lifetime of a WSN.

In previous research, a mobile sink [3] has been proposed to mitigate the above problem. In this method, a sink node can move and accordingly routes to the sink node and traffic flows change, distributing loads among sensor nodes. Nevertheless, a mobile sink has two problems. One is the overhead generated when nodes update routes to the sink node, and the other is the increase of a node’s active time in monitoring aperiodic queries from the sink node. The first problem can be partly solved, e.g., by using asymmetric communication [4]. However, nodes must be active at least when receiving the routing information from the sink node. The second problem can be partially solved, e.g., by using wake-up control [5]. In the case of multi-hop communication, each sensor node needs to send a wake-up message to activate its relay node on the communication route. Because an ordinary communication module is often used for transmitting the wake-up message, this leads to large power consumption.

This paper proposes an energy-efficient data collection method where sink-based centralized transmission scheduling (SC-Sched) is realized by integrating asymmetric communication and Wake-up Radio (WuR). This is based on the fact that the sink node and other sensor nodes are not the same. A sink node, typically mounted on a vehicle, has a stable power supply (using a rechargeable vehicle battery with a much larger capacity) unlike sensor nodes. Therefore, the sink node can use a large amount of power to transmit messages, directly reaching sensor nodes distributed in a wide area without relaying. Together with the wake-up control, it enables the sink node to directly control each sensor node. In this way, the proposed SC-Sched method reduces node active time by activating a sensor node only for data collection. In addition, it solves the problems of a mobile sink and improves network lifetime by exploiting sink mobility.

Specifically, in the proposed method, sensor nodes do not update routing information according to the position of the sink node. Instead, the sink node collects node position information, and on this basis determines a tree-based routing and a transmission order. In addition, each sensor node is equipped with a wake-up receiver (WuRx). At the time that a packet is to be exchanged between two sensor nodes, the sink node transmits a wake-up message using a transmission power larger than that used for data transmission, directly activating both transmitting and receiving sensor nodes simultaneously to start the communication. Here, the communication path between the sink node and sensor nodes is asymmetric, one-hop in the downlink (from the sink node to sensor nodes), and multi-hop in the uplink (from sensor nodes to the sink node). In the initial experiments, it is noticed that severe packet loss occurs in the presence of multipath fading, and the data collection time is long due to delay occurring at the time of activating the transmitting and receiving sensor nodes by a wake-up message per hop. Therefore, this paper further introduces a retransmission mechanism to deal with frame loss, and reduces data collection time by using different frequency channels for wake-up messages and data transmission.

We implemented the proposed SC-Sched method in the network simulator ns-2 using the parameters of a real WuRx and a data transceiver. Evaluations show that: (i) It is possible to shorten data collection time (caused by wake-up latency) by more than 31% via using multiple channels; (ii) Compared with the conventional duty-cycling method, SC-Sched greatly reduces total energy consumption, by up to 96.1% in a WSN with 86 nodes; (iii) Network lifetime can be improved further, by up to 28.2%, when the number of data collection points is increased from 1 to 4 in the presence of sink mobility; and (iv) SC-Sched prolongs the network lifetime by 7.47 times compared with the conventional duty-cycling method when the number of collection points is 4 in a WSN with 300 sensor nodes.

The contribution of this paper is fourfold, as follows:

- We propose an energy-efficient data collection method by integrating asymmetric communication and WuR. In the proposed method, the activation timing and routing of each sensor node are controlled by wake-up calls (WuCs) from the sink node. Thereby, each sensor node’s active time is reduced to the minimum.
- The proposed method has an adaptive retransmission mechanism to deal with frame loss of both a WuC and a data frame, which greatly improves system reliability.
- This paper further proposes to transmit WuCs and data frames on different channels in parallel, which helps to reduce data collection time.
- All proposed methods are implemented on a network simulator (ns-2) and evaluated by extensive simulations in different respects, e.g., network lifetime, the impact of sink mobility, and comparison with the conventional duty-cycling method.

I and II were reported in a conference paper [6]. III is newly added, and IV is greatly enhanced in this paper.

The rest of this paper is organized as follows. Section 2 describes related work. Section 3 explains the proposed method and Section 4 presents the simulation evaluation. Finally, Section 5 concludes this paper and points out future work.

## 2. Related Work

Here, related work on duty cycling, mobile sinks, asymmetric communication, and wake-up control is briefly reviewed.

### 2.1. Duty Cycling

A typical problem in WSNs is energy waste due to idle listening. Duty cycling is a conventional method to solve this problem. To reduce idle listening, each sensor node usually is in the sleep state and periodically wakes up to detect communication demands.

Different variants of duty cycling have been proposed [7]. In X-MAC [8], a sender node periodically transmits a short preamble carrying the receiver address until the receiver node wakes up, detects the preamble, and responds with an acknowledge frame (ACK). After receiving the ACK, the sender node transmits the data frame. Instead of using a preamble in X-MAC, in ContikiMAC [9] a sender node periodically transmits the data frame until it receives an ACK from the receiver or reaches the upper limit of retransmission.

In these duty-cycling media access control (MAC) protocols, nodes are not synchronized to a common wake-up schedule, and a large communication delay tends to occur. This delay can be shortened by increasing the duty cycle (the percentage of active time) at the cost of more power consumption. In addition, repeated transmissions of a single message more than once at the sender node also lead to energy consumption.

### 2.2. Mobile Sink

Another problem in WSNs is the hot-spot issue, in other words, sensor nodes near the sink node tend to drain their battery more quickly than other nodes. A mobile sink is an effective solution to this problem. In mobile sink methods [3], a sink node can move in the network. Then, routes between sensor nodes and the sink node change accordingly, which distributes the load of packet forwarding among sensor nodes. Nevertheless, a mobile sink introduces two new problems. One is routing overhead. Here, each sensor node maintains a route to the sink node and this route needs to be updated when the sink node changes its position. The other is the increase of a node’s active time in monitoring aperiodic queries from the sink node. With a fixed sink and periodic data collection, each sensor node can perform sleep/wake-up scheduling to save power. However, with a mobile sink, each sensor node does not know when a query from the sink node will come and needs to be awake for a longer time.

### 2.3. Asymmetric Communication

**Figure 1:**
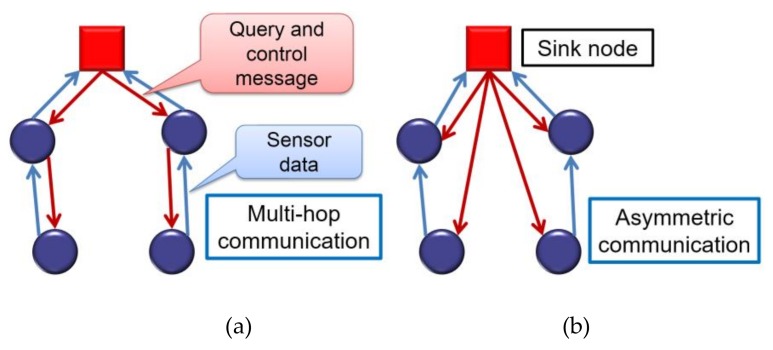
Communication paths: (**a**) symmetric multi-hop communication and (**b**) asymmetric communication.

Typically, all nodes in a WSN transmit with the same power. Then, communications between the sink node and sensor nodes are symmetric in either direction (uplink or downlink), and both require multiple hops as shown in Figure 1a.

As explained in Section 1, a sink node usually has a stable power supply compared with sensor nodes, which enables asymmetric communication [4] (Figure 1b). Here, a sink node with a stable power supply can transmit with a larger power and directly reach all sensor nodes. In contrast, sensor nodes transmit with a small amount of power and reach the sink node by multi-hop routes. In this way, asymmetric communication helps to reduce the power consumption at each sensor node in downlink transmissions. However, most transmissions in WSNs are uplink. Therefore, applying asymmetric communication alone will not largely contribute to the reduction of energy consumption.

### 2.4. Wake-Up Radio

In remote wake-up control [5], a WuRx, which consumes much less power than a sensor node, is used to control the wake-up timing of a sensor node. This helps reduce each sensor node’s active time and energy consumption as well. The wake-up control message is called a wake-up call (WuC). A WuC contains the address of the sensor node to be activated. When a WuRx receives a WuC indicating a communication request, it checks the address in the WuC by comparing it with the sensor node’s own address, and if matched, activates the sensor node to communicate. The study in [10] shows that using WuR is more energy efficient than duty-cycling MAC protocols (e.g., X-MAC [8] and RI-MAC [11]) and ensures a real-time response as well.

### 2.5. A Comparison of the Proposed Method and Related Works

The proposed method adopts WuR [5] instead of duty cycling [7] for power saving by reducing the sensor node’s active time. Also, a mobile sink [3] is adopted to distribute traffic load. However, the proposed method solves the problems of a mobile sink by integrating asymmetric communication [4] and WuR. Aperiodic queries are monitored with a low-power WuRx. The proposed method also reduces the overhead of updating routing information by transmitting a WuC to activate sensor nodes per hop. By asymmetric communication, a WuC directly reaches all sensor nodes, and each sensor node only needs to receive a WuC from the sink node and does not need to relay the WuC.

## 3. Sink-Based Centralized Transmission Scheduling

We propose an energy-efficient data collection method, sink-based centralized transmission scheduling (SC-Sched), realized by integrating asymmetric communication and WuR in order to solve the two problems of a mobile sink.

### 3.1. Overview of the Proposed Method

The key idea of SC-Sched is that the sink node performs transmission scheduling and uses asymmetric communication and WuR to directly activate sensor nodes involved in the communication just before the scheduled transmission timing. It works as follows.

1. The sink node decides the routing and transmission timing of each sensor node. When the sink node tries to collect sensor data, it transmits a WuC to directly activate a pair of sensor nodes per hop on the route by asymmetric communication.
2. Sensor nodes stay in the sleep mode and monitor aperiodic queries with their WuRxs. A WuRx receiving a WuC that matches its own address activates its sensor node.

**Figure 2:**
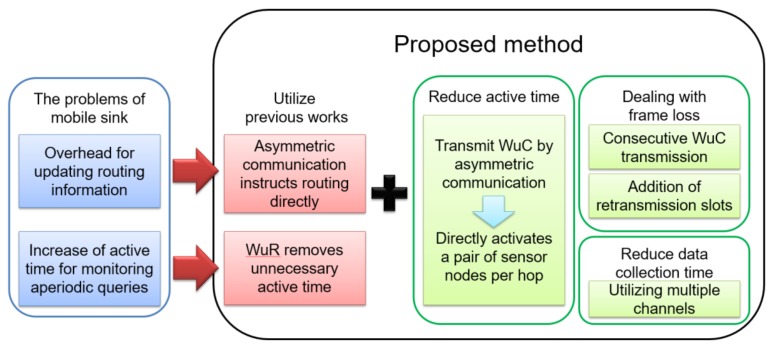
Conceptual diagram of the proposed method. WuR = wake-up radio; WuC = wake-up call.

Figure 2 is a conceptual diagram illustrating the components of the proposed method. Besides the asymmetric communication and wake-up control mentioned in previous works, the SC-Sched method introduces three new functions. Firstly, to reduce the active time of sensor nodes, the sink node directly activates a pair of sensor nodes per hop by integrating asymmetric communication and WuR. Second, to deal with frame loss, consecutive WuC transmission and an addition of retransmission slots are adopted. Finally, to reduce data collection time, by using different channels for WuC and data transmission, activation of sensor nodes and data transmission are made in parallel to mitigate the effect of delay generated when activating a pair of sensor nodes by a WuC per hop.

In the following, Section 3.2 explains the node model, Section 3.3 describes the basic data collection procedure where a sink node utilizes WuCs to wake up sensor nodes per hop. Section 3.4 explains how to deal with frame loss caused by multipath fading. Section 3.5 further suggests shortening data collection time by using multiple channels.

### 3.2. Node Model

A sensor node consists of a data communication module, a Micro Controller Unit (MCU), and a low-power WuRx. The data communication module and the WuRx both work in the 920 MHz band, which has less interference and higher reachability compared with the 2.4 GHz ISM band. Due to the hardware constraint, there is a wake-up latency [12] during which a node transits from sleep to the active state but cannot transmit or receive data. There is also a delay in detecting a WuC, which is called the WuC detection time [13]. This is the time taken for a WuRx to match its own address with the address in a WuC. Generally, the circuit configuration and performance limit the ability to achieve low energy consumption in the WuRx. Therefore, the WuC detection time is relatively long.

A sensor node can be in one of the six following states, where transition, idle, receiving, and transmitting are defined as active states.

- Sleep: The communication module and the MCU are in the sleep mode. A WuRx is active to monitor for WuCs.
- Detecting WuC: A WuRx is receiving a WuC and matching it with its own address.
- Transition: The main part of a sensor node (the MCU and the communication module) transits from sleep to the active state.
- Idle: Both the MCU and the communication module are active, but not involved in transceiving.
- Receiving: The communication module is receiving a data frame or an ACK frame.
- Transmitting: The communication module is transmitting a data frame or an ACK frame.

### 3.3. Data Collection Procedure

The sink node stops at each data collection point, computes routes based on the basic geographic routing [14], and decides the transmission order as follows:

1. The sink node calculates the distance between itself and each sensor node (the location of each sensor node is assumed to be known).
2. For each sensor node, treat the nodes that are in its transmission range as its neighbors.
3. For each sensor node, select from its neighbors the node that is the closest to the sink node as its relay node.
4. A tree structure is constructed with the sink node as the root node.
5. A depth-first search is executed on the tree. The result of the post-order is the transmission order.

Then, at each data collection point, the sink node collects sensor data from all sensor nodes by repeating the following steps.

1. In accordance with the transmission order, the sink node transmits a WuC per hop, which carries two addresses, one deciding the sender of data and the other deciding the receiver, where the receiver is actually the relay node for the sender.
2. WuRxs at the sender and the receiver, on receiving the WuC, activate their sensor nodes, which communicate during the time (called the communication possible time) assigned by the sink node.
3. After the communication possible time is completed, both the sender and receiver nodes enter the sleep mode again.
4. After the communication possible time, the sink node transmits a WuC to activate the next pair of nodes.

Whenever the sink node moves to a new data collection point, it recalculates routes and the transmission order based on the position of the data collection point before collecting sensor data.

The communication possible time is defined in terms of slots. The number of slots in a communication possible time is equal to the number of data frames (including frames to be forwarded) at a sender node. The definition of a slot is given by Equation (1).

(1) $$\begin{array}{ll} 1\;\mathrm{slot} & =\mathrm{data}\;\mathrm{frame}\;\mathrm{transmission}\;\mathrm{time}\\ & +\max\;\mathrm{propagation}\;\mathrm{delay}\;\mathrm{for}\;\mathrm{data}\;\mathrm{frame}\;\left(4\text{µ}s\right)\\ & +\mathrm{SIFS}\;\left(10\text{µ}s\right)\\ & +\mathrm{ACK}\;\mathrm{frame}\;\mathrm{transmission}\;\mathrm{time}\\ & +\max\;\mathrm{propagation}\;\mathrm{delay}\;\mathrm{for}\;\mathrm{ACK}\;\mathrm{frame}\;\left(4\text{µ}s\right) \end{array}$$

where SIFS (short inter-frame space) is the space separating a data frame and its ACK.

**Figure 3:**
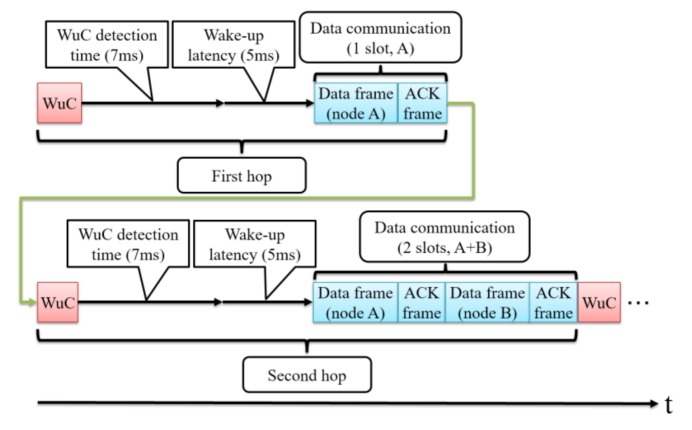
Time-sequence diagram of the data collection procedure.

**Figure 4:**
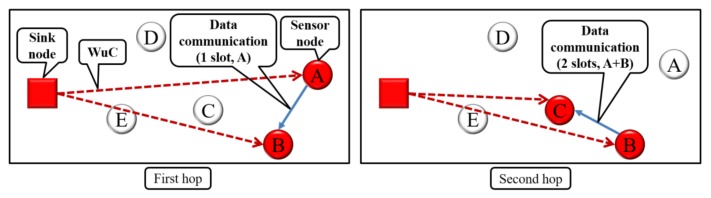
Data collection procedure in the proposed method.

Figure 3 and Figure 4 show an example of the data collection procedure. First, a WuC is sent to activate A and B, and one frame is transmitted from A to B (first hop). Then, a WuC is sent to activate B and C, and two frames are transmitted from B to C (second hop).

### 3.4. Dealing with Frame Loss

In real environments, signal strength dynamically fluctuates due to multipath fading, which leads to frame loss and degrades the frame delivery ratio. In SC-Sched, this loss is composed of two parts: one is the loss of WuCs, and the other is the loss of data frames.

In the following, we improve SC-Sched to cope with both loss factors by using consecutive WuC transmissions and adding retransmission slots for data frames. WuCs are the one-way traffic over the asymmetric link and cannot be acknowledged. Therefore, we use consecutive transmissions to reduce the loss of WuCs.

#### 3.4.1. Consecutive WuC Transmission

If a sensor node misses a WuC, the node cannot wake up to transmit its data frame. The function of consecutive WuC transmissions reduces the probability that a sensor node will fail to wake up.

WuC retransmission timing is determined by considering the WuC detection time. In order to wake up both the sender and the receiver at the same time, we add a sequence number to each WuC. For example, in the case of three transmissions, the sequence number is changed in the following order: 2, 1, 0. Both nodes check when the retransmission of the WuC will end (the sequence number becomes 0) by referring to the sequence number and decide the wake-up timing.

The number of WuC transmissions is determined by the sink node according to the following algorithm.

1. Calculate the average Signal-to-Noise Ratio (SNR) based on the distance between the sink node and the sender node in this hop.
2. Determine the WuC error rate (WER) from the average SNR using the SNR-BER (Bit Error Rate) table and the property of channel fading.
3. Find *n*, the minimal number of WuC transmissions that satisfies the following condition: ${\mathrm{WER}}^{n}\;<0.0025$, i.e., the target WER under retransmissions is no more than 0.25%.

#### 3.4.2. Addition of Retransmission Slots for Data

**Figure 5:**
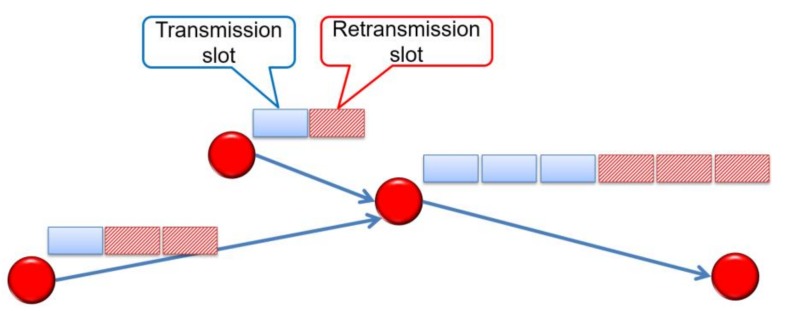
Conceptual diagram of the retransmission slots for data frames.

If a sender node retransmits a data frame when it fails to receive an ACK frame, there will be a shortage in the communication possible time. This is because the number of slots assigned as the communication possible time is equal to the number of data frames owned (and to be forwarded) by the sender without considering retransmissions. Hence, the sender node cannot finish transmitting all data frames whenever retransmissions occur. To solve this problem, we add retransmission slots to the default communication possible time. An example of assigning retransmission slots is shown in Figure 5.

The number of retransmission slots is determined by the sink node according to the following algorithm.

1. Calculate the average SNR based on the distance between the sender and the receiver of a hop.
2. Determine the slot error rate (SER) from the average SNR using the SNR-BER table and the property of channel fading.
3. Calculate the minimal number of retransmission slots *n* so that all data frames at a sender node, sharing *n* retransmission slots, are transmitted successfully with a probability no less than 99.75%, in other words, the frame error rate under retransmissions is no more than 0.25%.

### 3.5. Using Multiple Channels for Shortening Data Collection Time

In the SC-Sched method, the sink node activates a pair of sensor nodes per hop. When a sensor node wakes up, the sensor node performs WuC detection and address matching and activates the data module for transmitting/receiving. These operations take a relatively long time and occur every hop as shown in Figure 3. Therefore, the SC-Sched method takes a relatively long time to collect all sensor data.

We suggest using multiple channels to shorten the data collection time of SC-Sched, where the WuC and data frames are transmitted over different channels in parallel. This adopts the concept of pipelined wake-up [13], but all of the transmissions are controlled by the sink node.

**Figure 6:**
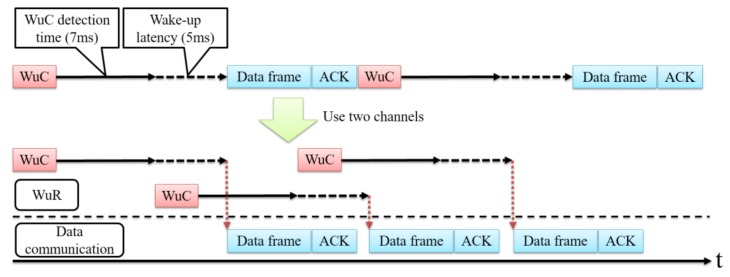
Reducing data collection time by parallel transmission of WuCs and data frames over multiple channels.

Figure 6 shows the mechanism of using multiple channels. The next WuC can be transmitted immediately after the WuC detection time. When the number of data frames relayed by a node is large, it is possible that the transmission of the next pair of nodes will start before the transmission of the current pair is finished, and the two transmissions will collide with each other. To prevent this problem, the sink node schedules the transmission timing of the next WuC based on the calculated data transmission completion time. In this way, the next pair of nodes wake up properly, neither too early (with transmission collision) nor too late (with extra waiting, which increases data collection time), and the transmission of the next data frame exactly follows the current one.

## 4. Simulation Evaluation

We implemented the proposed SC-Sched method on the network simulator ns-2 [15] and compared it against a method WuR-TDMA, which implements part of the functions of the proposed method, but uses different channel-access methods. Furthermore, we simulated ContikiMAC [9] on the network simulator ns-3 [16] for a comparison.

### 4.1. Simulation Setup

#### 4.1.1. Comparison Method

WuR-TDMA: The sink node broadcasts a WuC to activate all sensor nodes at once. Then, the sink node broadcasts the information of each node’s routing and transmission timing by the data communication module using a large amount of power to reach all sensor nodes. Each sensor node communicates by TDMA (time-division multiple access) based on the received information. More specifically, after receiving the routing/transmission timing information from the sink node, each sensor node enters the sleep mode and schedules to wake up on its own time slots to transmit/forward its packets. Unlike SC-Sched, WuR-TDMA has the overhead of notifying sensor nodes that there is information (activation timing, communication possible time, and routing) for sensor data transmission. In order to verify the effect of reducing this overhead, SC-Sched is compared with WuR-TDMA.

ContikiMAC [9]: ContikiMAC is one of the well-known duty-cycling MAC protocols commonly used in WSNs. A node periodically wakes up to detect potential communication demands (receive packets). A sender node periodically transmits its data frame until it receives an ACK from the receiver or reaches the upper limit of the number of retransmissions. SC-Sched is compared with ContikiMAC to show the effectiveness of avoiding periodical wake-up by applying WuR.

Table 1 shows the main differences between the SC-Sched method and the two comparison methods. In the simulation of ContikiMAC, it is assumed that the routing is already known.

#### 4.1.2. Simulation Scenario

We evaluated all three methods in two scenarios, fixed sink and mobile sink. In both scenarios, one data collection is defined as a process during which the sink node collects sensor data from all sensor nodes once. Each sensor node generates one data frame for each data collection. When the number of sensor nodes is 86 or 196, the sensor nodes are arranged in a grid shape at intervals of 150 m in times of 86 nodes, and 100 m in times of 196 nodes. As for other cases, sensor nodes are randomly distributed in the communication range corresponding to the maximal power of the sink node.

In the fixed sink scenario, the sink node stays at the center of the simulation area and collects data once. Three hundred simulations with random seeds are performed and the average results are computed.

In the mobile sink scenario, there are four data collection points: (400, 0), (800, 400), (400, 800), and (0, 400). The sink node stays at one to four points with rotation per data collection. The movement pattern is as follows: (400, 0) for one point, (400, 0), (400, 800) for two points, (400, 0), (400, 800), (0, 400) for three points, and (400, 0), (800, 400), (400, 800), (0, 400) for four points. The simulated time is 24 h and the sink node collects data once per hour.

#### 4.1.3. Simulation Parameters

Simulation parameters are shown in Table 2. The data frame size is set to 50-byte with a 17-byte header and 33-byte sensor data. In [17], a sensor node generates 3-byte sensor data per sensing, namely, each data frame contains the temperature and humidity data of 11 sensings.

Table 3 shows a sensor node’s power consumption at each state decided by [18,19,20]. Note that the power consumption of WuRx is excluded in ContikiMAC.

#### 4.1.4. Evaluation Metric

The following metrics are common to both scenarios.

- Total energy consumption: Total energy consumed by all sensor nodes in one data collection.
- Frame delivery ratio: The number of data frames reaching the sink node divided by the number of data frames that should reach the sink node, where the latter is equal to the number of sensor nodes.
- The following metric is only used for the fixed sink scenario.
- Data collection time: The period from the time the sink node transmits the first WuC to the time the sink node returns the ACK frame for the last-received sensor data.
- The following metrics are only used for the mobile sink scenario.
- Maximum energy consumption: Energy consumption of the node with the highest energy consumption within 24 h.
- Network lifetime: The time when first dead node appears (Computed by dividing the initial energy (10,000 (J)) with the maximum energy consumption).

In the following, Section 4.2.1 and Section 4.2.2 describe the evaluation of the two functions of SC-Sched in the fixed sink scenario, respectively. Section 4.2.3 shows the performance of SC-Sched in the mobile sink scenario. Section 4.3 compares the performance of the three methods in the two scenarios.

### 4.2. Evaluation of SC-Sched

#### 4.2.1. Evaluation of Dealing with Frame Loss

Here, we evaluate the function of consecutive transmissions and retransmissions.

In Section 3.4, the number of WuC transmissions and the number of data retransmission slots are made adaptive to average SNR. As a comparison, we also evaluate another method, which is SC-Sched with fixed retransmission policies regardless of SNR. In this simulation, the number of sensor nodes is 600 and we do not use multiple channels.

Table 4 shows the effect of the number of WuC transmissions and retransmission slots on three metrics.

We first focus on the case of “Fixed”. The frame delivery ratio increases with the number of WuC transmissions and data retransmission slots. On the other hand, total energy consumption and data collection time increase as well. That is, there is a trade-off between frame delivery ratio and both total energy consumption and data collection time.

Next, the cases of “Adaptive” and “Fixed” are compared. When the number of WuC transmissions is 6 and the number of retransmission slots is 3, the frame delivery ratio of “Fixed” is lower than that of “Adaptive”. Nevertheless, the total energy consumption and data collection time of “Adaptive” are much less than those of “Fixed”. In summary, deciding the number of WuC transmissions and data retransmission slots adaptively per hop is effective in improving the frame delivery ratio while suppressing total energy consumption and data collection time.

#### 4.2.2. Evaluation of Using Multiple Channels

Here, we evaluate the effect of using multiple channels. Table 5 shows the results of three metrics and the reduction ratio of data collection time. The reduction ratio is defined as 1 − *r*, where *r* is the ratio of data collection time with multiple channels to that without multiple channels.

By using multiple channels, the data collection time is reduced by over 31% irrespective of the number of nodes. Currently, one channel is used for WuCs and another channel is used for data frames. In the future, we plan to further reduce data collection time by increasing the number of channels used for data communication.

#### 4.2.3. Result of Mobile Sink Scenario

Using a mobile sink is one of the features of SC-Sched. Here, we evaluate the effect of traffic load distribution on network lifetime in the mobile sink scenario.

Table 6 shows the simulation results of four metrics and the improvement in network lifetime (compared to the network lifetime when the number of data collection points is fixed to 1). As the number of data collection points increases, the maximum energy consumption is decreased. In addition, the improvement in network lifetime increases with the number of nodes. This indicates that when the node density is high, SC-Sched can distribute the traffic load to more nodes and better balance the traffic by exploiting sink movement.

### 4.3. Comparison with Other Methods

Here, we evaluate the superiority of SC-Sched by comparing it with the other two methods in the two simulation scenarios. Additionally, in the fixed sink scenario, we also evaluate the basic SC-Sched method (called SC-Sched(simple)), which does not have the functions of retransmissions for dealing with frame loss and parallel transmissions on different channels for shortening data collection time. Note that the parameters of ContikiMAC are adjusted so that it achieves a similar frame delivery ratio to SC-Sched while keeping its energy consumption is as low as possible (the duty cycle is 5%, the retransmission limit of data frames is 4, and the contention window size is set according to the node density).

#### 4.3.1. Result in the Fixed Sink Scenario

**Figure 7:**
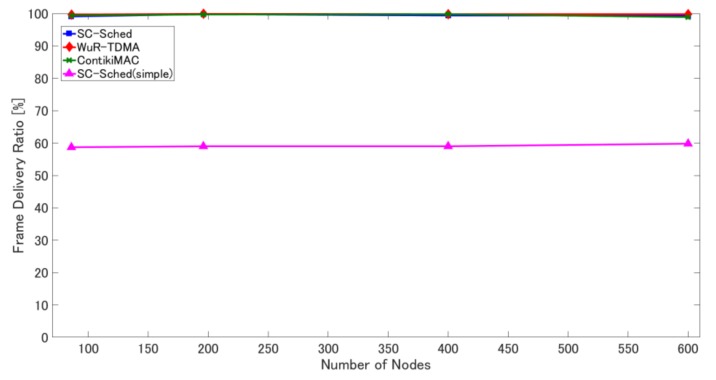
Frame delivery ratio in the four methods under different numbers of sensor nodes.

**Figure 8:**
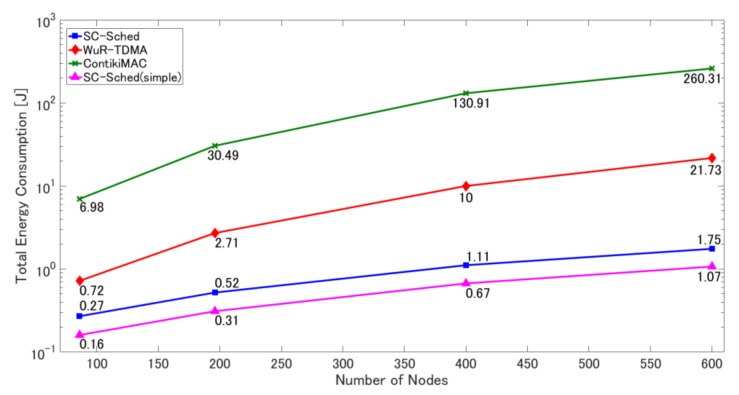
Total energy consumption in the four methods under different numbers of sensor nodes.

**Figure 9:**
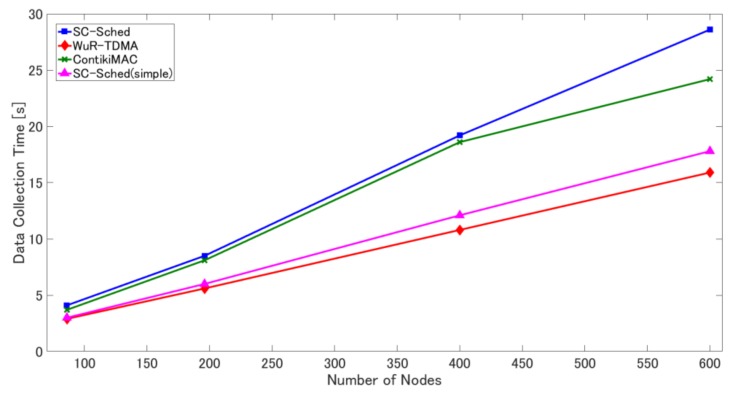
Data collection time in the four methods under different numbers of sensor nodes.

Figure 7, Figure 8 and Figure 9 show the simulation results of the four methods for each metric in the fixed sink scenario. As shown in Figure 7, SC-Sched(simple) without retransmission mechanisms has a very low frame delivery ratio, about 60%, which confirms the necessity of adopting retransmission mechanisms in SC-Sched.

As shown in Figure 8, the total energy consumption of SC-Sched is much lower than that of WuR-TDMA and ContikiMAC. In WuR-TDMA, receiving from the sink node the information of each node’s routing by the communication module causes much overhead, so the active time of all sensor nodes is prolonged and total energy consumption increases. In ContikiMAC, there are four overheads that affect active time. First, the sender uses CSMA/CA and it is necessary to wait for a random backoff time before actual transmissions. Second, the sender repeats transmission of the data frame until an ACK is received from the receiver. Third, a surrounding node, in its active period, may overhear a data frame not addressed to itself. Finally, each sensor node needs to wake up periodically by duty cycling. In comparison, SC-Sched achieves much lower power consumption by removing all of these overheads. The total energy consumption of SC-Sched(simple) is lower than that of SC-Sched, because in times of a reception failure of a WuC, the sensor nodes remain in the sleep state. This is achieved at the cost of a low frame delivery ratio.

However, as shown in Figure 9, the data collection time of SC-Sched, after using multiple channels, is still longer than that of the other methods. In SC-Sched, the sender node and the receiver node are woken up by a WuC per hop, and the WuC detection time and wake-up latency of the data communication module occur at each hop. By using multiple channels, the transmission (and detection) of a WuC and wake up of the data communication module for the next hop can be done in parallel with the data transmission of the current hop. For the pairs of leaf nodes in the routing tree there is only one data packet, and its transmission time is shorter than the overall time of wake-up latency of the data communication module and the WuC detection time in the presence of consecutive WuC transmissions. Therefore, the data collection time of SC-Sched is prolonged. In ContikiMAC, WuC detection time does not exist and wake-up latency does not influence the data collection time by early wake up. However, there is still a wait time due to CSMA/CA and duty cycling. In contrast, in WuR-TDMA, all sensor nodes are woken up at once. Therefore, the WuC detection time and wake-up latency occur only once, and the data collection time of WuR-TDMA is the shortest among the four methods. However, this is achieved at the cost of more energy consumption in receiving the routing and transmission scheduling information from the sink node. The data collection time of SC-Sched(simple) is lower than that of SC-Sched because SC-Sched (simple) has no retransmissions for WuCs and data frames.

#### 4.3.2. Result in the Mobile Sink Scenario

**Figure 10:**
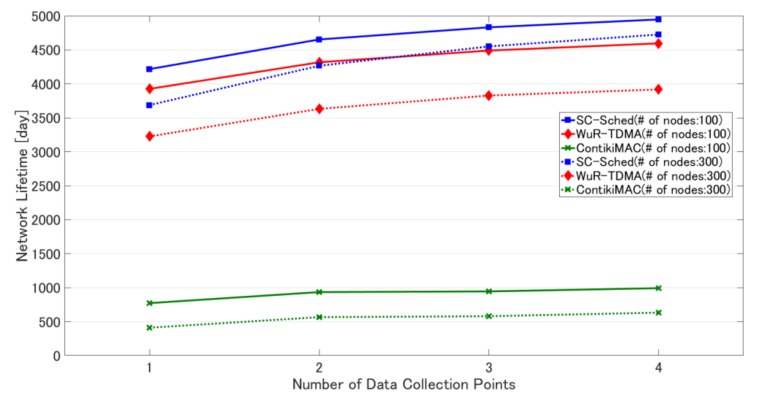
Network lifetime in the three methods under different numbers of data collection points.

Table 7 shows the simulation results of the three methods in the mobile sink scenario. The results on network lifetime when the number of nodes is 100 and 300 are shown in Figure 10.

Table 7 indicates that as the number of data collection points increases, the maximum energy consumption decreases. As a result, the network’s lifetime is prolonged. The network’s lifetime is the longest when collecting at four points using SC-Sched regardless of the number of nodes. In WuR-TDMA, as mentioned in Section 4.3.1, energy consumption due to receiving routing and transmission timing information is large, and the amount of routing and transmission timing information increases in proportion to the number of nodes. Therefore, the improvement in network lifetime is higher in SC-Sched than in WuR-TDMA, and the difference increases with the number of nodes. These results are also visually shown in Figure 10.

Compared with the result in Figure 8, the difference in network lifetime between SC-Sched and WuR-TDMA may seem less than expected. This is because the difference in Figure 8 only occurs during the data-collection process without considering the power consumption in the long sleep period. In the mobile sink scenario, the sink node collects data 24 times during 24 h, and the energy consumption in the sleep state accounts for the majority of the total energy consumption. Hence, the power difference between WuR-TDMA and SC-Sched in the data-collection process is not very obvious and has little impact on network lifetime. Because WuR-TDMA implements part of the function of SC-Sched, this also confirms the effectiveness of SC-Sched. When the data collection frequency increases, the percentage of power consumption due to data collection will increase and SC-Sched will show more superiority.

In SC-Sched, the improvement in network lifetime in times of 300 nodes is higher than that in times of 100 nodes. This means that the higher the number of nodes, the higher the effect of traffic load balancing by sink movement.

### 4.4. Brief Summary

In SC-Sched, a sink node decides the communication schedule and precisely controls the wake-up timing of each sensor node. This helps reduce the loss due to collisions in CSMA/CA and the idle listening time. Furthermore, a sink node includes the routing information in WuCs, which greatly reduces the overhead of updating routing information in the mobile sink scenario. Therefore, SC-Sched can benefit from more data collection points with little overhead and improve network lifetime. By activating nodes only for actual communication without idle listening, SC-Sched reduces energy consumption by almost one order compared with ContikiMAC. However, its data collection time, after using two channels, is still longer than that of the other methods. The question of how to further reduce the data collection time is left to future work.

## 5. Conclusions

In this paper, we proposed an energy-efficient data collection method for large scale WSNs, where Sink-based Centralized transmission scheduling (SC-Sched) is realized by integrating asymmetric communication and wake-up radio. In SC-Sched, sensor nodes do not update routing information. Instead, a sink node collects node position information and on this basis determines a tree-based routing and a transmission order. Each sensor node is equipped with a wake-up receiver. At the time that a packet is to be transmitted between two sensor nodes, the sink node transmits a Wake-up Call (WuC) using a transmission power larger than that used for data transmission, directly activating both transmitting and receiving sensor nodes simultaneously to start the communication. WuC includes routing and communication possible time determined by the sink node. In this way, the sink node directly controls each sensor node to reduce the sensor node’s active time.

Simulation results of SC-Sched show that (i) the algorithm for adaptively determining the number of WuC transmissions and data retransmission slots per hop helps achieve a high frame delivery ratio while suppressing the total energy consumption and data collection time, and (ii) it is possible to shorten the data collection time by more than 31% by using different channels for WuC and data transmission.

We compared SC-Sched with WuR-TDMA (where the sink node broadcasts the routing information and transmission timing information to all nodes at once) and ContikiMAC (a conventional duty-cycling method). Simulation results show that SC-Sched reduces the total energy consumption by 62.5% compared with WuR-TDMA and by 96.1% compared with ContikiMAC when the number of sensor nodes is 86. This is because SC-Sched reduces each sensor node’s active time to the minimum: it is active only for data collection, which avoids the overhead of updating the routing information and duty cycling. Moreover, SC-Sched prolongs network lifetime by 7.47 times compared with ContikiMAC when the number of sensor nodes is 300 and the number of data collection points is 4.

In the future, we will further reduce the data collection time. We will also try to extend SC-Sched to cope with an increase in network scale and node failure and improve the routing construction algorithm by considering better traffic load distribution and the residual energy of each sensor node.

## Figures and Tables

**Table 1:** Comparison of different methods.

Method	SC-Sched	WuR-TDMA	ContikiMAC
Activation timing	A pair of nodes per hop	All nodes at once firstly	Duty cycling
Routing and transmission timing information	WuC per hop	All together by communication module	Routing is known
Channel access	TDMA	TDMA	CSMA/CA ^*^

^*^ CSMA/CA: Carrier-sense multiple access with collision avoidance

**Table 2:** Simulation parameters.

Parameter	Value	
Method	SC-Sched, WuR-TDMA	ContikiMAC
Network simulator	ns-2.34	ns-3.26
Number of sensor nodes	86/196/400/600 (fixed sink scenario), 100/200/300 (mobile sink scenario)	
WuC size	11 byte	
Data frame size	50 bytes (frame header 17 bytes + sensor data 33 bytes)	
Frequency band	920 MHz	
Bit-rate	100 Kbps	
Modulation	OOK (WuRx), GFSK (comm. module)	
Propagation model	Friis + Nakagami	Two Ray Ground
Area	1600 m × 1600 m (fixed sink scenario), 800 m × 800 m (mobile sink scenario)	
Positon of sink node	Center of area (fixed sink scenario), Midpoint of each side (mobile sink scenario)	
Transmission range	800 m (sink), 200 m (sensor)	
Transmission power	24 dBm (sink), 0 dBm (sensor)	
WuC detection time	7 ms	-
Wake-up latency	5 ms	
Simulation times	300 (fixed sink scenario), 100 (mobile sink scenario)	

**Table 3:** A sensor node’s power consumption at each state.

State	Power Consumption (WuRx + Communication Module + MCU)
Sleep	20.7 µW (2.4 µW + 3.0 µW + 15.3 µW)
Detecting WuC	25.5 µW (7.2 µW + 3.0 µW + 15.3 µW)
Transition	24.4 mW (2.4 µW + 19 mW + 5.4 mW)
Idle	57.2 mW (2.4 µW + 57 mW + 163.5 µW)
Receiving	62.4 mW (2.4 µW + 57 mW + 5.4 mW)
Transmitting	74.4 mW (2.4 µW + 69 mW + 5.4 mW)

WuRx = wake-up receiver; MCU = micro controller unit.

**Table 4:** Evaluation of dealing with frame loss.

Number of WuC Transmissions		Number of Retransmission Slots		Frame Delivery Ratio (%)	Total Energy Consumption (J)	Data Collection Time (s)
Fixed	4	Fixed	1	95.5	1.82	35.0
	4		2	96.7	1.86	37.9
	4		3	96.8	1.90	40.8
	5		1	96.7	1.89	39.7
	5		2	98.0	1.96	42.6
	5		3	98.3	2.00	45.6
	6		1	97.2	1.97	44.4
	6		2	98.6	2.02	47.4
	6		3	99.0	2.07	50.3
Adaptive		Adaptive		99.4	1.94	41.8

**Table 5:** Evaluation of using multiple channels.

Number of Nodes	Multiple Channels	Frame Delivery Ratio (%)	Total Energy Consumption (J)	Data Collection Time (s)	Reduction Ratio (%)
86	None	99.1	0.27	6.2	33.9
	Use	99.1	0.27	4.1	
196	None	99.7	0.54	13.8	38.4
	Use	99.8	0.52	8.5	
400	None	99.4	1.20	27.9	31.2
	Use	99.4	1.11	19.2	
600	None	99.4	1.94	41.8	31.6
	Use	99.4	1.75	28.6	

**Table 6:** Evaluation of SC-Sched in the mobile sink scenario.

Number of Nodes	Number of Data Collection Points	Frame Delivery Ratio (%)	Total Energy Consumption (J)	Maximum Energy Consumption (J)	Network Lifetime (day)	Improvement in Network Lifetime (%)
100	1	99.6	184.2	2.39	4217.2	-
	2	99.8	184.2	2.15	4653.8	10.4
	3	99.9	184.2	2.07	4832.8	14.6
	4	99.8	184.2	2.02	4948.3	17.3
200	1	99.6	368.0	2.57	3922.8	-
	2	99.8	368.1	2.26	4429.5	12.9
	3	99.8	368.1	2.15	4662.0	18.8
	4	99.8	368.1	2.07	4824.1	23.0
300	1	99.6	552.0	2.75	3687.7	-
	2	99.8	552.0	2.35	4266.6	15.7
	3	99.9	552.0	2.20	4551.5	23.4
	4	99.9	552.0	2.12	4726.6	28.2

**Table 7:** Evaluation in the mobile sink scenario.

Number of Nodes	Method	Number of Data Collection Points	Frame Delivery Ratio (%)	Total Energy Consumption (J)	Maximum Energy Consumption (J)	Network Lifetime (day)	Improvement in Network Lifetime (%)
100	SC-Sched	1	99.6	184.2	2.39	4217.2	17.3
		4	99.8	184.2	2.02	4948.3	
	WuR- TDMA	1	99.9	198.1	2.56	3926.5	17.1
		4	99.9	198.2	2.18	4596.4	
	Contiki MAC	1	99.8	844.2	13.31	754.9	28.9
		4	99.7	843.2	10.29	972.9	
200	SC-Sched	1	99.6	368.0	2.57	3922.8	23.0
		4	99.8	368.1	2.07	4824.1	
	WuR- TDMA	1	99.9	421.7	2.87	3500.9	21.0
		4	99.9	421.8	2.36	4236.4	
	Contiki MAC	1	99.9	1987.6	20.39	495.7	48.5
		4	99.9	1985.2	13.60	736.2	
300	SC-Sched	1	99.6	552.0	2.75	3687.7	28.2
		4	99.9	552.0	2.12	4726.6	
	WuR- TDMA	1	99.8	671.7	3.12	3226.2	21.4
		4	99.9	671.7	2.55	3918.2	
	Contiki MAC	1	99.1	3262.8	24.59	410.8	54.1
		4	99.2	3260.9	15.83	632.9	
